# Promising immunotherapeutic approaches for primary effusion lymphoma

**DOI:** 10.37349/etat.2024.00242

**Published:** 2024-06-26

**Authors:** Jutatip Panaampon, Seiji Okada

**Affiliations:** IRCCS Istituto Romagnolo per lo Studio dei Tumori (IRST) "Dino Amadori", Italy; ^1^Division of Hematologic Neoplasia, Department of Medical Oncology, Dana-Farber Cancer Institute, Boston, MA 02215, USA; ^2^Department of Medicine, Harvard Medical School, Boston, MA 02215, USA; ^3^Division of Hematopoiesis, Joint Research Center for Human Retrovirus Infection, Kumamoto University, Kumamoto 860-0811, Japan

**Keywords:** Primary effusion lymphoma, immunotherapeutic approaches, immunotherapy

## Abstract

Primary effusion lymphoma (PEL) is a large B-cell neoplasm usually presenting as a serious effusion in body cavities without detectable tumor masses. It is an AIDS-related non-Hodgkin’s lymphoma (HL) with human herpes virus 8 (HHV8)/Kaposi sarcoma-associated herpes virus (KSHV) infection. A combination antiretroviral therapy (cART) prolongs the lifespan of AIDS and AIDS-related malignant lymphoma patients, but PEL continues to have a dismal prognosis. PEL showed disappointing outcomes with standard chemotherapy such as CHOP or CHOP-like regimens. A PEL status highlights the urgent need for new therapeutic approaches and treatment strategies and improve clinical outcomes. This review discusses the current knowledge and some recent clinical trials for PEL in the platform of immunotherapy as well as promising future immunotherapeutic approaches for PEL.

## Introduction

Primary effusion lymphoma (PEL) is a rare B-cell malignant lymphoma with malignant effusions without tumor mass [1]. Recently, PEL forming solid tumor mass, named extracavitary PEL, is also described [2]. It was first described in 1989 as an AIDS-related lymphoma [3]. It is always associated with human herpes virus 8 (HHV8)/Kaposi sarcoma-associated herpes virus (KSHV) infection, and often occurs in immunodeficient states such as AIDS [4–6]. PEL resists standard chemotherapeutic regimens such as CHOP (cyclophosphamide, doxorubicin, vincristine, and prednisone) or CHOP-like regimens. PEL patients have a very poor prognosis and a short median survival of less than 1 year even in the current era of effective combination therapy [2, 7]. New therapeutic approaches for PEL are urgently needed to improve the prognosis.

HHV8/KSHV was identified in 1994 in an HIV-positive patient with acquired immune deficiency syndrome and Kaposi sarcoma [8]. HHV8 is associated with B-cell lymphomas including multicentric Castleman disease (MCD) [9], Kaposi sarcoma, diffuse large B-cell lymphoma (DLBCL) [10], and PEL [4]. MCD is an unusual systemic lymphoproliferative disease and includes a form associated with KSHV/HHV8 and includes immunocompromised status such as HIV infection [11]. HHV8/KSHV is an etiologic agent for PEL, with approximately 80% of cases having Epstein-Barr virus (EBV) coinfection with latency I [12]; however, the requirement for EBV coinfection is not clear. Recently, it has been shown that EBV supports the KSHV persistence and EBV nuclear antigen 1 (EBNA-1) increases KSHV viral load and latency-associated nuclear antigen-1 (LANA-1) expression, indicating that EBV co-infection supports KSHV tumorigenesis [13, 14].

Immunotherapies for malignant diseases have developed rapidly in recent decades. Immunotherapy has become a leading area, seeing numerous ongoing studies and promising immunotherapeutic approaches against malignancies. Herein, we review the current knowledge of immunotherapies and immunotherapeutic approaches for clinical PEL treatment as well as PEL clinical trials. In addition, we will briefly highlight promising future immunotherapeutic approaches for PEL hematopoietic malignancies.

### PEL and its immunophenotypic features

According to the 4th edition of the WHO Classification of Tumors of Hematopoietic and Lymphoid Tissues, PEL is classified as a large B-cell neoplasm featuring serious effusions without tumor masses, and is universally associated with HHV8/KSHV. PEL is discussed in both the 5th edition of the WHO manuscript [15] and the International Consensus Classification (ICC) [16]. As for the differential diagnosis, there exist neoplasms featuring serious effusion such as diffuse large cell lymphoma and Burkitt’s lymphoma with effusion [17]. Recently classified HHV8 negative PEL-like lymphoma shows similar clinical and pathological features (lymphomatous effusion without detectable mass), except for being not infected HHB8/KSHV and CD20 positive, termed “HHV8/KSHV negative effusion-based lymphoma” was proposed as another category of lymphoma [18, 19]. Immunohistochemical staining of HHV8 LANA-1 is used for the diagnosis of PEL. The genome of HHV8/KSHV is about 145 kb. PEL cells contain 40–80 copies of HHV8/KSHV episomes per cell and positive for latent gene expression including LANA-1, LANA-2/viral interferon-regulatory factor-3 (vIRF-3), viral cyclin (v-cyclin), viral FLICE inhibitory protein (v-FLIP) and Kaposin (K12) [17]. In most cases (50–80%), EBV is co-infected; however, the role of EBV for PEL tumorigenesis is still not clear. There are 50–80% PEL with EBV co-infection. EBNA-1 and Epstein-Barr virus-encoded small RNA (EBER), which are stated in latency I of EBV infection are considered positive in PEL [4, 17].

PEL morphologies show large cells with plasmablastic/immunoblastic/anaplastic cytology. The pathological examination and diagnosis for PEL is basically done by cytologic preparation of effusion fluid. PEL cells display large nuclei with round or irregular morphology while the basophilic cytoplasm [7, 17].

**Figure 1:**
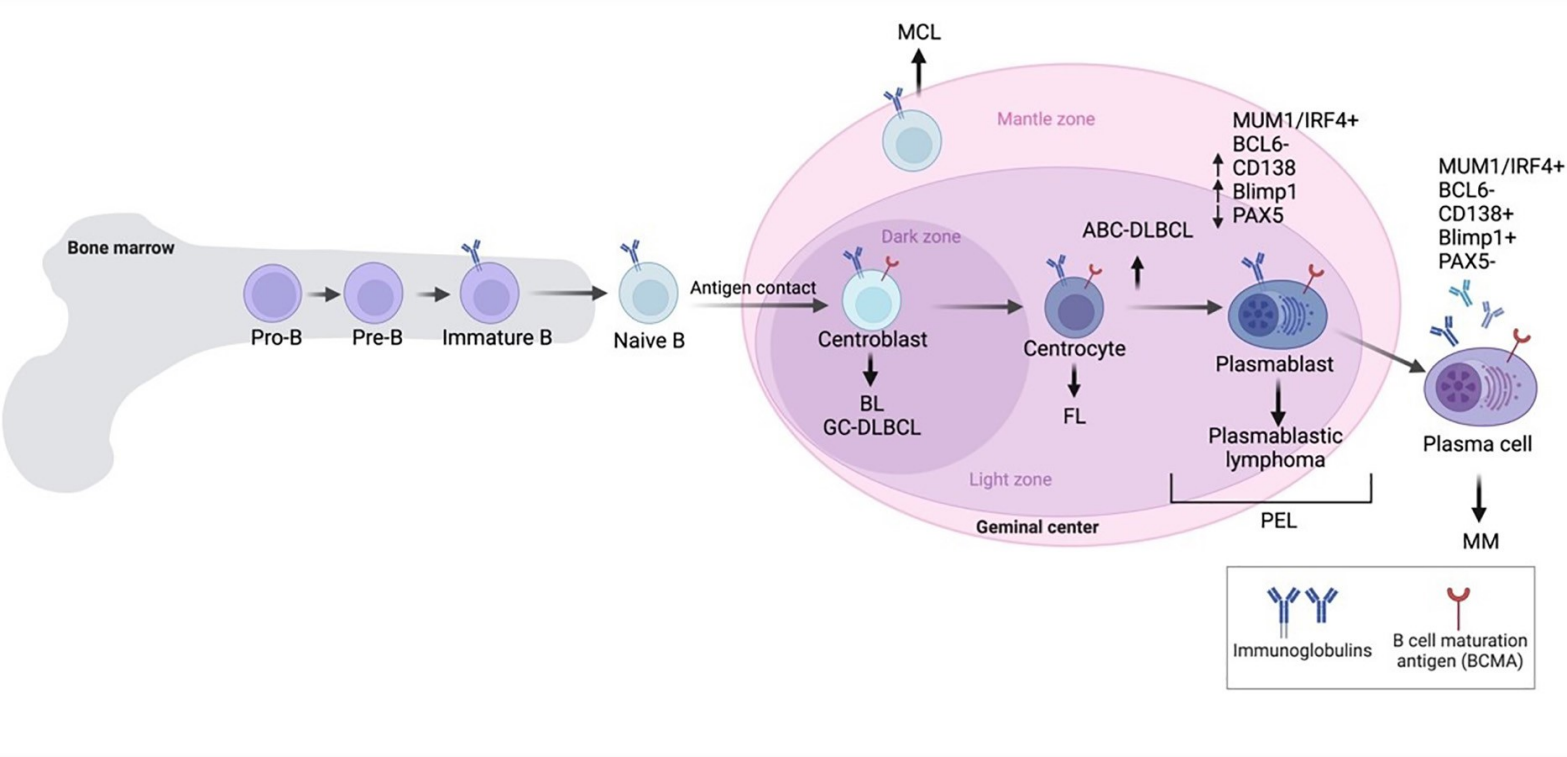
An overview of B cell malignancies in each stage of B-cell development. ABC-DLBCL: activated B-cell subtype diffuse large B cell lymphoma; BL: Burkitt’s lymphoma; FL: follicular lymphoma; GCB-DLBCL: germinal center B-cell subtype diffuse large B cell lymphoma; IRF4: interferon regulatory factor 4; MCL: mantle cell lymphoma; MM: multiple myeloma; PEL: primary effusion lymphoma; MUM1: multiple myeloma oncogene-1. Created with BioRender.com

PEL is typically positive for CD45 leukocyte marker without expression of T cell markers such as CD2, CD3, CD4, CD5, CD7, and CD8, and absence of *Bcl-6* and *c-myc* [17]. PEL is a B-cell malignancy but is generally negative for B-cell markers such as CD19, CD20, CD79a, surface and cytoplasmic immunoglobulin [20, 21]. Rare B cell non-Hodgkin lymphoma (NHL), which is negative for CD20 expression are approximately 1–2% of overall B cell lymphomas. CD20-negative B cell NHL often have aggressive characteristics and is resistant to standard chemotherapy, leading to a very poor prognosis. PEL expresses CD38, CD30, CD138/syndecan-1 molecule, epithelial membrane antigen (EMA), multiple myeloma oncogene-1 (MUM1)/interferon regulatory factor 4 (IRF4), and human leukocyte antigen-DR (HLA-DR) [6]. The phenotypic profile of PEL coupled with immunogenotypic features, indicates that PEL is in an advanced state of B-cell differentiation as a plasmablastic derivation/pre-plasma cells (Figure 1) [6].

## Immunotherapeutic approaches for PEL treatment

Over the past few decades, numerous studies have focused on advanced immunotherapies against hematologic malignancies and cancer, making significant clinical progress, especially in immunotherapy for hematologic malignancies. Advanced cell-based immunotherapies such as chimeric antigen receptor (CAR) T cell therapy and monoclonal antibody (mAb) have been approved by the United States Food and Drug Administration (FDA) for clinical use. Various studies in the immunotherapy field are at pre-clinical and clinical trial stages.

## mAbs

mAbs exhibit therapeutic efficacy against cancerous cells by targeting specific surface antigens or overexpressed antigens on tumor cells [22]. Antibody-based strategies highlight the enhanced anti-tumor responses of effector immune cells, modulating multiple mechanisms including antibody-dependent cell cytotoxicity (ADCC), antibody-dependent cell phagocytosis (ADCP), and effector immune cell-independent mechanisms such as an antibody-direct effect to induce cell death and complement-dependent cytolysis (CDC). ADCC is the mechanism by which, depending on Fc receptor (FcR)-bearing effector immune cells and tumor antigen binding by the Fab portion of mAb, the release of cytotoxic granules including granzyme and perforin is triggered to kill malignant cells. Natural killer (NK) cells are innate immune cells and play critical roles in immune surveillance. CD107a is a common functional marker for the identification of NK cell activity [23].

### Rituximab: anti-CD20 mAb

Rituximab is an mAb targeting CD20 approved by FDA in 1997. Since then, rituximab has become a standard mAb for B-cell malignancy treatment regimens. FDA has approved rituximab for a variety of B-cell malignancies, as shown in Table 1.

**Table 1:** FDA approval of rituximab for use against B-cell malignancies [24, 25]

**CD20 positive B-NHL**	**CLL**
Single agent to treat relapsed/refractory, low grade, or follicular B cell NHL	Combination with fludarabine and cyclophosphamide
Single agent therapy for those who achieve a complete or some degree of response to combination chemotherapy	
Single agent in low grade stable B cell NHL after CVP chemotherapy	
Combination with anthracycline-based chemotherapy in untreated DLBCL	

B-NHL: B cell non-Hodgkin’s lymphoma; CLL: chronic lymphocytic leukemia; DLBCL: diffuse-large B cell lymphoma

Rare B cell NHL without CD20 expression accounts for approximately 1–2% of overall B cell lymphomas. CD20-negative B cell NHL frequently shows an aggressive clinical manifestation tolerant to standard chemotherapy, and has a very poor prognosis. The most common CD20-negative B cell NHLs are PEL, large B-cell lymphoma arising from HHV8/KSHV-MCD, plasmablastic lymphoma (PBL), and ALK-positive large B cell lymphoma [26]. MCD is an unusual systemic lymphoproliferative disease and includes a form associated with KSHV/HHV-8 and including immunocompromised status such as HIV infection [11]. Rituximab was an efficacious treatment for patients with MCD, reducing inflammatory symptoms and cytokine levels and prolonging survival [27, 28]. Although PEL is a CD20-negative B-cell malignancy, many PEL patients have concurrent KSHV-MCD for which rituximab is the standard treatment. Rituximab likely kills KSHV-infected CD20-positive B cells in germinal centers that are a source of inflammatory cytokines. Two HIV-infected patients with MCD who received etoposide therapy and four infusions of rituximab developed Kaposi sarcoma with virologic relapses as an increase of blood HHV8 DNA [29]. Nevertheless, considering its effect, rituximab may help to eliminate the KSHV CD20 positive B-cell reservoir that produces inflammatory cytokines and triggers PEL initiation and progression in patients with PEL who have concurrent MCD [12].

Phase I [30] and I/II [31] clinical studies are monitoring and evaluating the efficacy of rituximab in patients with PEL and MCD under the hypothesis that rituximab eradicates circulating KSHV-infected B cells and reduces KSHV viremia and inflammatory cytokines.

### Daratumumab (anti-CD38 mAb)

**Figure 2:**
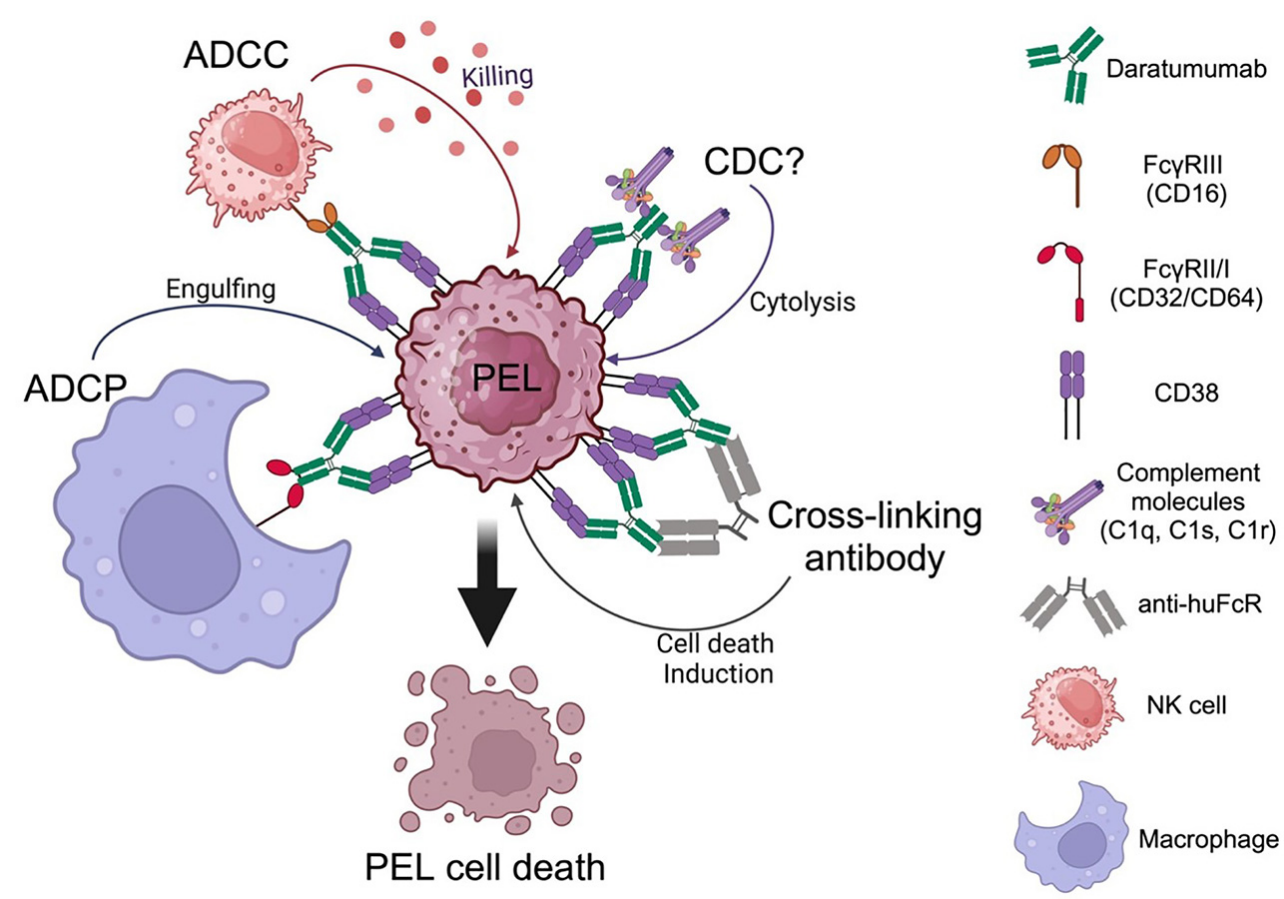
Daratumumab mediates the killing of primary effusion lymphoma (PEL) via multiple mechanisms. ADCC: antibody-dependent cell cytotoxicity; ADCP: antibody-dependent cell phagocytosis; CDC: complement-dependent cytolysis; NK: natural killer. Created with BioRender.com

CD38 is reliably expressed on PEL [7, 32–34]. Daratumumab is a fully human mAb, targeting CD38 and approved by the FDA for multiple myeloma (MM). Daratumumab possesses multiple mechanisms against PEL. Shrestha et al. [34] and our pre-clinical study [32] demonstrated that daratumumab confers potent efficacies as therapeutic mAb against PEL cells by mediating ADCC. In addition, we showed that daratumumab confers CDC, ADCP and cell death induction by cross-linking [32]. We reported that NK cells demonstrated killing activity against PEL and that daratumumab significantly enhanced NK cytotoxicity via ADCC. Daratumumab also elevated phagocytotic activity by elevating the percentage of PEL-engulfing macrophages. By employing rabbit serum as a source of complement, our study indicated that daratumumab confers CDC against PEL whereas the study by Shrestha and colleagues which utilized human serum as a source of complement showed that daratumumab did not induce CDC activity for PEL [34] due to PEL having a high complement of inhibitory molecules including CD55 and CD59, which are species-specific [35, 36]. The mechanism of daratumumab against PEL is shown in Figure 2. Most PEL cell lines and PEL cells from patients highly express CD38, yet some PEL cell lines express lower CD38 on their surfaces. The potent activity of mAb to increase ADCC activity depends on the antigen expression on target cell surfaces [37, 38]. ADCC induction by daratumumab in MM and CLL cells correlates with the level of surface CD38 expression [39, 40]. All-trans retinoic acid (ATRA) increases CD38 expression on PEL and enhances daratumumab-mediated ADCC induction [34]. Daratumumab single agent treatment demonstrated a potent inhibitory effect on PEL cell growth in PEL xenograft immunodeficient mice [32].

A case report of one HIV-infected patient with PEL in 2018 demonstrated successful clinical benefits in relapsed PEL [33]. This patient received dose-adjusted rituximab, etoposide, prednisone, vincristine, and doxorubicin; brentuximab vedotin (anti-CD30 mAb drug conjugate); and gemcitabine with oxaliplatin followed by ifosfamide, cyclophosphamide, and etoposide. At day 100 the patient showed evidence of relapsed PEL together with recurrent HHV8 viremia. The patient subsequently received 16 mg/kg daratumumab weekly resulting in a reduction of HHV8 viral titer from 807,000 copies/mL to an undetectable level after the first dose. Moreover, CT imaging showed no evidence of lymphoma at approximately three months from the start of daratumumab administration [33].

Another case report of one elderly PEL patient with HIV negative status in 2021 demonstrated successful clinical benefits of the use of daratumumab in relapsed PEL as a prolonged clinical remission for twelve months at the time of the case report and maintenance with good quality of life without adverse effects from the therapy [41]. The patient received 16 mg/kg daratumumab monotherapy weekly for eight weeks, followed by a dose every two weeks for four months and monthly thereafter. Polymerase chain reaction (PCR) could detect no HHV8 in the patient’s blood following the first dose of daratumumab administration. After four months of daratumumab treatment, PET imaging showed a good metabolic response and a decline in pericardial effusion without evidence of fluorodeoxyglucose (FDG) avid lymphoma [41].

In contrast to rituximab’s side effect, daratumumab does not result in normal B cell depletion. Daratumumab should be considered for future clinical trials for PEL and MCD. Lurain K., a principal investigator from the USA National Cancer Institute, is leading clinical trials to evaluate daratumumab in relapsed/refractory PEL (NCT5907759).

### Elotuzumab (anti-SLAMF7 mAb)

**Figure 3:**
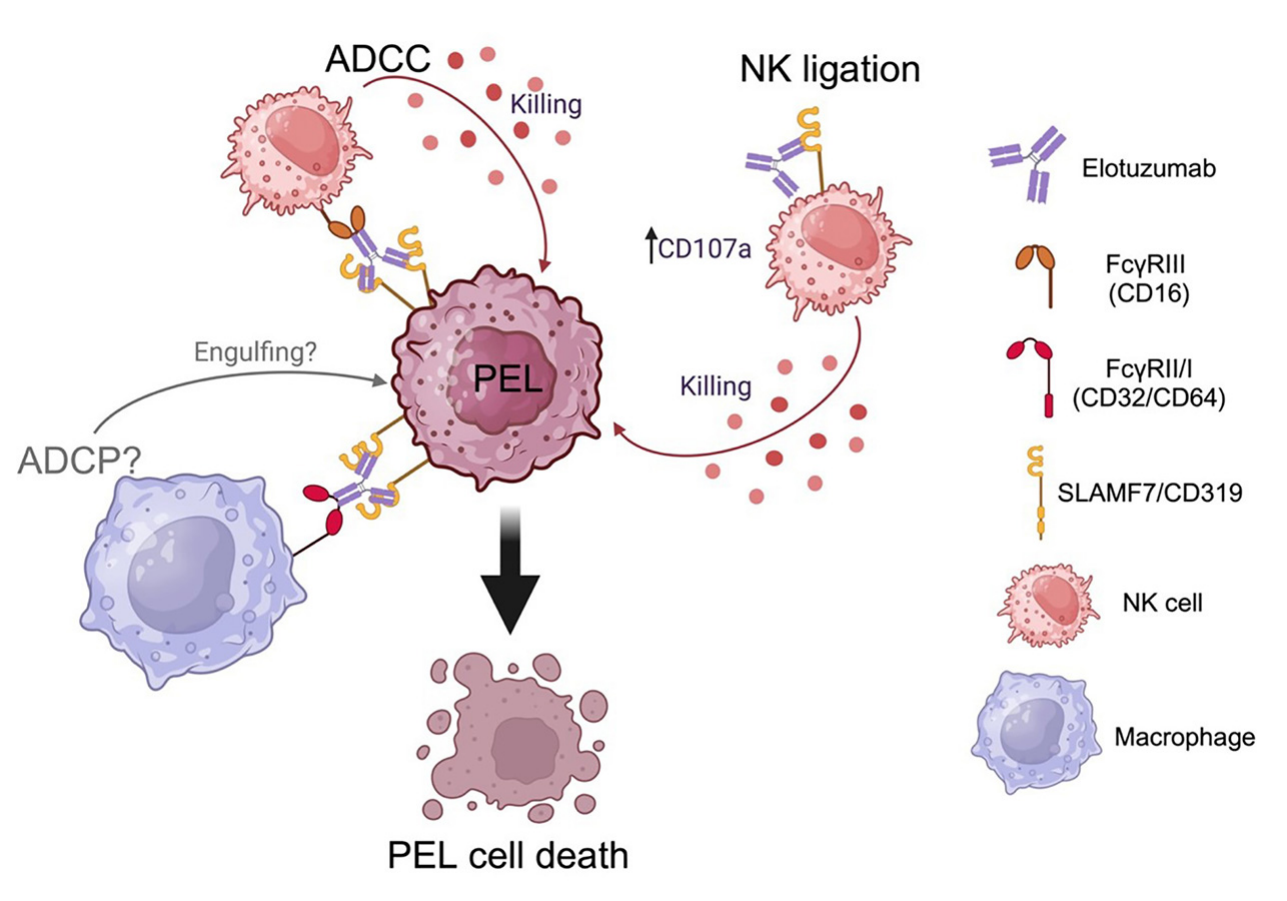
Elotuzumab mediates the killing of primary effusion lymphoma (PEL) via multiple mechanisms. ADCC: antibody-dependent cell cytotoxicity; ADCP: antibody-dependent cell phagocytosis; NK: natural killer; SLAMF7: signaling lymphocytic activation molecule 7. Created with BioRender.com

SLAMF7 (signaling lymphocytic activation molecule 7), also known as CD319, CS1, or CRACC, is notably expressed on PEL [42]. A study in patient samples demonstrated that SLAMF7 highly expresses on PEL [43, 44] and is a potential diagnostic marker in PBL and BC-2, which is a PEL-derived cell line [44]. Elotuzumab is a humanized mAb, targeting SLAMF7 and approved by the FDA for MM. Elotuzumab binds to constant Ig-linked extracellular domains, resulting in the enhanced cytolytic function of NK cells and ADCC activities [45–47], ADCP [48] against MM, and CS1/SLAMF7 ligation [49, 50]. Furthermore, elotuzumab has been shown to enhance NK cell activation and myeloma cell killing through interleukin-2 (IL-2) and tumor necrosis factor alpha (TNF-α) pathways [51]. Our previous study clearly showed that elotuzumab confers ADCC activity against PEL [42]. In addition, Pazina et al. [49], Collins et al. [50], and our previous study [42] demonstrated that elotuzumab directly triggers NK cell activation through SLAMF7 ligation. Direct engagement of the Fc portion containing elotuzumab is indispensable for the activation of NK cells. Evidence regarding elotuzumab mediating CDC is inconclusive [42, 46]. The mechanism of elotuzumab against PEL is shown in Figure 3. Elotuzumab prolonged the survival of PEL-xenograft immunodeficient mice that received NK adoptive transfer [42]. Considering the potential mechanism of elotuzumab against PEL, ADCP should be further studied in order to obtain pre-clinical evidence for PEL therapeutic mAb therapeutic approaches.

### Anti-CD47 mAb

Cluster of differentiation 47 (CD47) or an integrin-associated protein (IAP) is ubiquitously expressed on normal cells and upregulated in various malignancies including hematologic malignancies such as leukemia/lymphoma [52] and solid tumors such as ovarian cancer, breast cancer, colon cancer, bladder cancer, prostate cancer, glioblastoma, and hepatocellular carcinoma [53]. Signal regulatory protein alpha (SIRPα) is identified as the ligand of CD47 [54]. SIRPα expresses on the surface of phagocytic cells including macrophages and dendritic cells [54, 55]. Normal cells including red blood cells and hematopoietic stem cells express CD47 to protect themselves from phagocytosis [56]. Engagement of CD47 and SIRPα results in anti-phagocytotic activity, known as the “Don’t eat me” mechanism [57]. High CD47 expression in malignancies correlates with poor prognosis [53]. Disruption of CD47-SIRPα binding is one of the immunotherapeutic approaches to trigger phagocytosis and inhibit tumor growth [58, 59].

**Figure 4:**
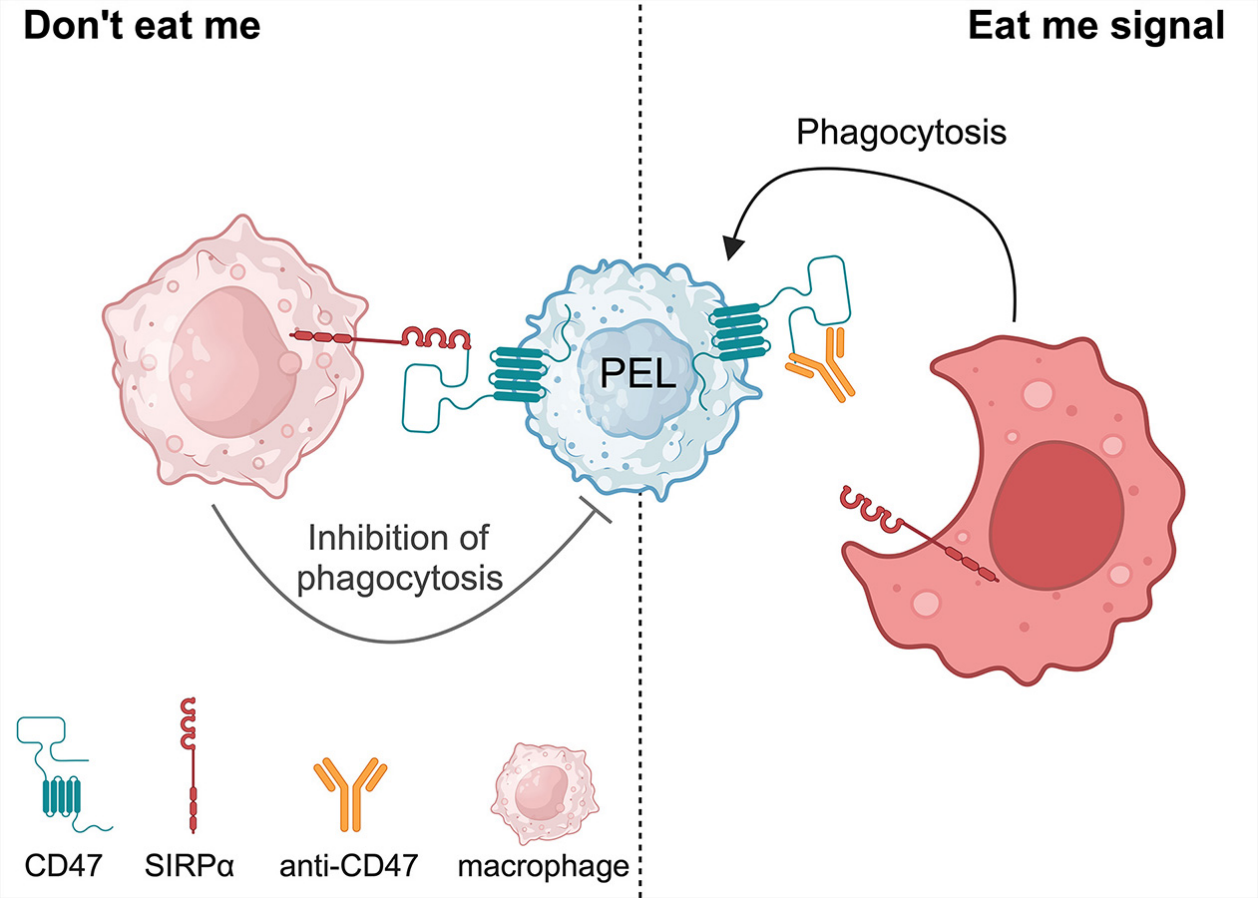
Anti-CD47 antibody promotes phagocytosis against primary effusion lymphoma (PEL). SIRPα: signal regulatory protein alpha. Created with BioRender.com

CD47 is expressed in many subsets of B-cell NHL including DLBCL, chronic lymphocytic leukemia (CLL), follicular lymphoma (FL), and mantle cell lymphoma (MCL) [60]. Anti-CD47 antibody blocks the binding of CD47-SIRPα and promotes phagocytosis [61]. Our previous study showed that CD47 is highly expressed on PEL cell lines [62]. We demonstrated that therapeutic CD47 knockdown in a PEL cell line enhanced phagocytosis of PEL in vitro. Moreover, anti-CD47 antibody is shown to promote phagocytosis in vitro. We used anti-CD47 antibody to treat PEL-xenografted mice, and the results indicated that anti-CD47 antibody inhibited ascites formation and organ invasion [62, 63]. Anti-CD47 antibody blocks the binding of CD47-SIRPα and promotes phagocytosis “Just eat me” against PEL (Figure 4).

## Antibody-drug conjugates

Antibody-drug conjugates (ADCs) are a type of mAbs covalently linked to a cytotoxic drug, providing wider possibilities to treat malignancies in terms of utilizing a highly specific targeting mAb together with a potent killing effect by a linked-cytotoxic drug. ADCs combine two powerful bullets: mAb and a cytotoxic agent. The mAb targets a specific malignant antigen while the cytotoxic agent is delivered precisely to kill malignancies. ADCs thus provide highly potent simultaneous cytotoxicity at a precise area based on the antigen-specificity of the tumor targets. Moreover, ADCs provide a wide therapeutic index because the toxicity on antigen-negative cells or normal tissues is limited [64]. ADC is a new type of anti-cancer drug, known as a “biological missile” [65]. FDA approved the first ADC, gemtuzumab ozogamicin, for the treatment of relapsed or refractory CD33-positive acute myeloid leukemia (AML) in 2000 [66]. Since then, numerous ADCs have been developed in order to achieve greater effectiveness for killing malignancies.

### Brentuximab vedotin (SGN-35)

**Figure 5:**
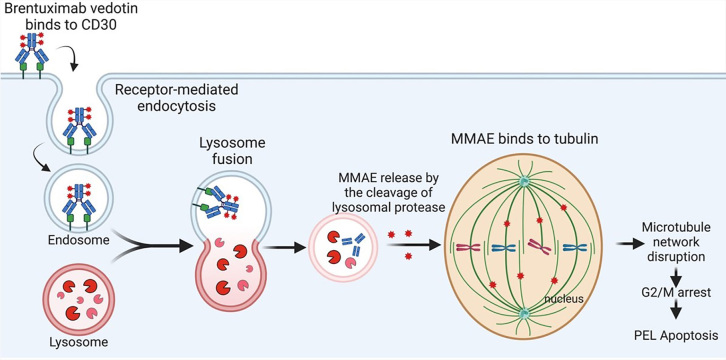
Brentuximab vedotin mediates the killing of primary effusion lymphoma (PEL). MMAE: monomethyl auristatin E. Created with BioRender.com

In 2011, FDA approved brentuximab vedotin (SGN-35) for treating relapsed Hodgkin’s lymphoma (HL) and systemic anaplastic larger cell lymphoma (ALCL) [67]. Brentuximab vedotin (SGN-35) is an anti-CD30 ADC that specifically targets CD30-positive cells and links them with an antimitotic agent, monomethyl auristatin E (MMAE). MMAE, a synthetic derivative of dolastatin 10, is an anti-tubulin agent [68]. Brentuximab vedotin binds to CD30 positive cells which then undergo internalization and endocytosis. Following digestion by endocytic enzymes, MMAE is released, binds to tubulin, and subsequently blocks tubulin polymerization and inhibits microtubule formation, resulting in the disruption of mitotic spindle assembly and cell cycle arrest [69]. Systemic delivery of MMAE itself is toxic as a single agent. By combining MMAE with a CD30-targeting antibody (brentuximab vedotin), clinical reports showed efficacy against lymphomas [67, 70–72]. CD30, also known as TNFSF8, belongs to the TNF receptor superfamily and is expressed in a small subset of activated B and T lymphocytes [73]. Classic HL and systemic ALCL express high levels of CD30 [74]. The majority of PEL have high CD30 expression [72]. A preclinical study indicated that brentuximab vedotin mono treatment reduced cell proliferation and induced cell cycle arrest at the G2/M phase and apoptosis in PEL cell lines [72]. In addition, in vivo brentuximab vedotin treatment inhibited tumor growth and prolonged the survival of PEL-bearing mice [72]. A recent case report in 2019 indicated that brentuximab vedotin treatment was efficacious in two PEL patients [75]. The study examined two patients with AIDS and PEL who received brentuximab vedotin for two cycles after disease refractory to dose-adjusted EPOCH (DA-EPOCH; etoposide, prednisone, vincristine, cyclophosphamide, doxorubicin). One patient showed complete remission for over 39 months whereas the other patient had new sites of disease with loss of CD30 expression [75], suggesting the need for combination treatment. Another case report in 2019 presented brentuximab vedotin and a combination antiretroviral therapy (cART) in a patient with HIV positive status associated with CD30-positive extra-cavitary PEL who achieved a durable complete response [76]. In 2021, a further case report noted a complete remission after successful brentuximab vedotin treatment in a non-HIV patient with relapsed PEL following CHOP regimen treatment [77]. As the patient had completed 16 cycles of brentuximab vedotin and exhibited significant clinical responses without toxicities, the clinicians decided to extend the treatment until any complications arose [77]. The mechanism of brentuximab vedotin for PEL killing is shown in Figure 5.

## Immunomodulatory agents

The immunomodulatory drugs (iMiDs) are a group of thalidomide derivatives such as lenalidomide, and pomalidomide. Lenalidomide and pomalidomide are used to treat MM. IMiDs target cereblon (encoded by the *CRBN* gene), which is an E3 ubiquitin ligase [78]. Cereblon is a substrate recognition subunit of cullin-RING E3 ubiquitin ligase complex CRL4^CRBN^. IMiDs-bound CRL4^CRBN^ complexes gain neo substrates [79–81], which provides anticancer activity. A study of MM revealed that lenalidomide causes selective ubiquitination and degradation of IKZF1 (Ikaros) and IKZF3 (Aiolos), which are lymphoid transcription factors, by the CRL4^CRBN^ ubiquitin ligase [80]. IRF4 is one of the downstream targets of CRBN, and is critical for MM survival. IRF4 is upregulated in PEL [6]. Moreover, IRF is down-regulated by iMiDs treatment [82]. IMiDs have a pronounced anti proliferative effect against PEL cell lines, causing cell cycle arrest at G0/G1 phase without reactivation of KSHV lytic replication [83]. It was reported in 2016 that iMiDs target the IKZF1-IRF4-MYC axis in PEL in a cereblon-dependent manner, while iMiDs mediate anti-PEL effects through cereblon-dependent suppression of IRF4 and subsequent IKZF1 degradation [83]. In addition, lenalidomide significantly prolonged the survival of PEL-bearing NOD/SCID mice compared with a vehicle control-treated group [83]. Moreover, combination treatment of lenalidomide and JQ-1, a BRD4 inhibitor, provided longer survival than lenalidomide treatment alone [83]. It is well known that KSHV infection results in major histocompatibility complex class 1 (MHC-I) downregulation during viral lytic replication. Furthermore, an intercellular adhesion molecule-1 (ICAM-1) and B7-2 (CD86) were downregulated in the KSHV latent infection state, enabling T cell and NK cell immune evasion, respectively. The study found that lenalidomide and pomalidomide prevented MHC-I downregulation during viral lytic activation, and restored ICAM-1 and B7-2 expression on the PEL cell surface in the viral latent infection state [84]. Thus, iMiDs reverse KSHV-induced downregulation of immune-surface markers and restore immune surveillance in PEL [84].

A prospective study with untreated PEL patients employed a regimen including a combination of lenalidomide with rituximab and dose-adjusted etoposide, prednisone, vincristine, cyclophosphamide, and doxorubicin (EPOCH; NCT02911142) [30]. A 50% complete remission rate and 66.7% 2-year overall survival were seen in six participants [30, 85]. A phase I/II study of lenalidomide combined with dose-adjusted infusion etoposide, vincristine, and doxorubicin with cyclophosphamide and prednisone (DA-EPOCH) and rituximab (DA-EPOCH-R^2^) in PEL patients is ongoing [31]. A retrospective study of patients with HIV-associated lymphomas including three PEL patients, and pembrolizumab (anti-PD1, a checkpoint blockage) therapy with or without pomalidomide revealed one PEL patient with a complete response and two other PEL patients with long-term disease control [86]. Lurain K. and colleagues are conducting a phase I clinical trial (NCT04902443) to evaluate the potential synergistic clinical responses of combined pomalidomide and nivolumab [85].

## Conclusions

**Figure 6:**
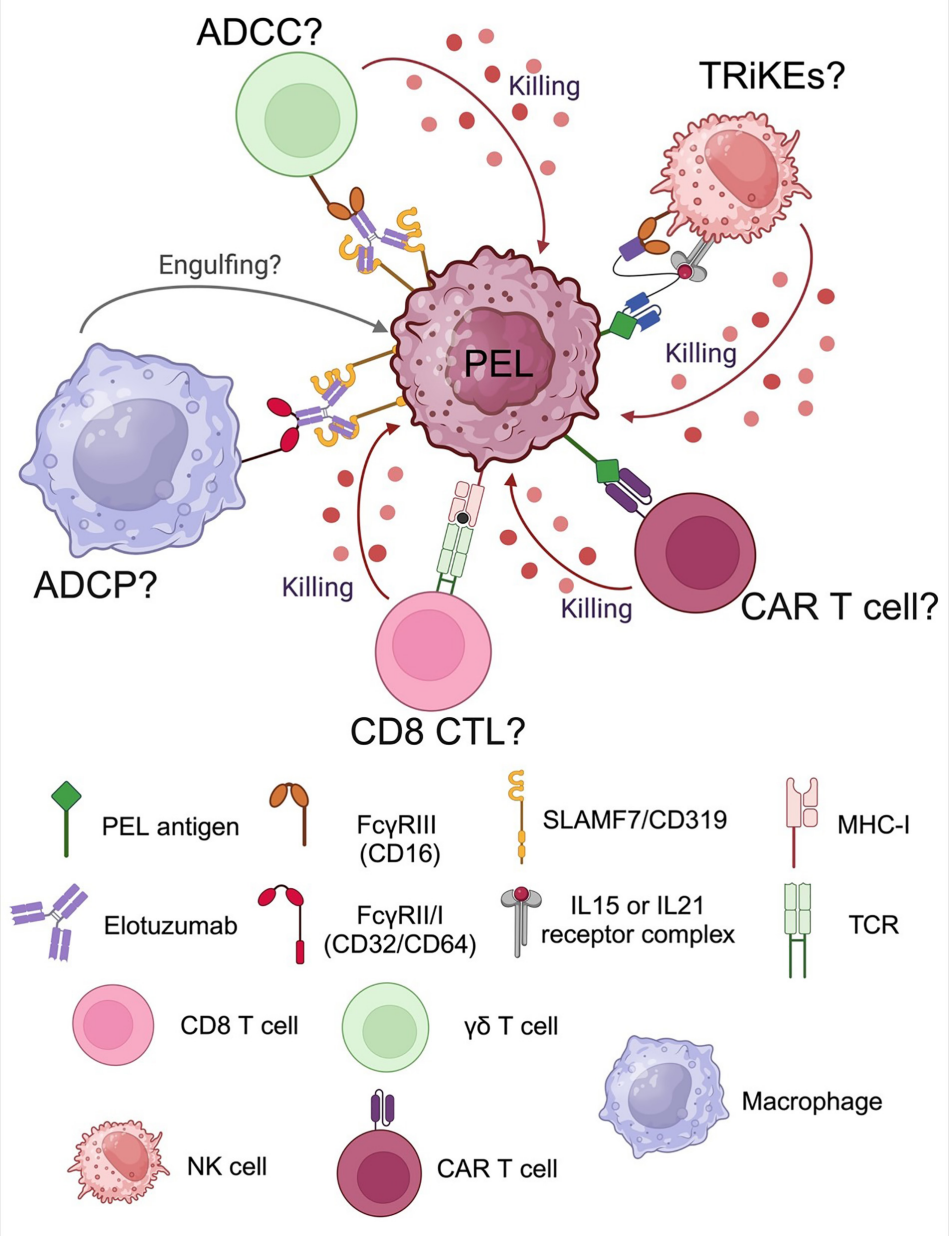
Future directions of promising immunotherapeutic approaches for primary effusion lymphoma (PEL). ADCC: antibody-dependent cell cytotoxicity; ADCP: antibody-dependent cell phagocytosis; CAR: chimeric antigen receptor; CTL: cytotoxic T lymphocytes; IL: interleukin; MHC- I: major histocompatibility complex class 1; NK: natural killer; SLAMF7: signaling lymphocytic activation molecule 7; TCR: T cell receptor; TRiKEs: tri-specific killer engagers. Created with BioRender.com

The schematic summary of future promising immunotherapeutic approaches for PEL is depicted in Figure 6. Using an antibody immunotherapy provides beneficial outcomes to enhance effectiveness of immune cells by ADCC and/or ADCP. Moreover, cell therapies should be considered for therapeutic approach such as cytotoxic T lymphocytes (CTL) or CAR T cells. Another approach is an engineering tool such as tri-specific killer engagers (TRiKEs) to lift up NK activity against PEL.

This is an exciting time in the field of immunotherapy for hematologic malignancies as well as advanced cell production for therapies [87]. The rarity of PEL cases makes it extremely challenging to develop new treatment strategies and conduct clinical trials. The discovery of broad and novel PEL-targeting antigens for immunotherapeutic approaches and preclinical evidence both in vitro and in vivo are critical prerequisites for future clinical treatments. In addition, the identification of any PEL antigens that are shared with other malignancies will permit utilization of the available FDA-approved targeting immunotherapies such as mAb or CAR T cells. We have been searching for PEL antigens as well as studying other effector immune cells to accumulate preclinical evidence for future PEL therapies.

## Abbreviations

ADCC: antibody-dependent cell cytotoxicity
ADCP: antibody-dependent cell phagocytosis
ADCs: antibody-drug conjugates
CAR: chimeric antigen receptor
CDC: complement-dependent cytolysis
CLL: chronic lymphocytic leukemia
DLBCL: diffuse large B-cell lymphoma
EBNA-1: Epstein-Barr virus nuclear antigen 1
EBV: Epstein-Barr virus
HHV8: human herpes virus 8
ICC: International Consensus Classification
iMiDs: immunomodulatory drugs
IRF4: interferon regulatory factor 4
KSHV: Kaposi sarcoma-associated herpes virus
LANA-1: latency-associated nuclear antigen-1
mAb: monoclonal antibody
MCD: multicentric Castleman disease
MHC: major histocompatibility complex
MM: multiple myeloma
MMAE: monomethyl auristatin E
NHL: non-Hodgkin lymphoma
NK: natural killer
PEL: primary effusion lymphoma
SIRPα: signal regulatory protein alpha
SLAMF7: signaling lymphocytic activation molecule 7
TNF: tumor necrosis factor

## Declarations

### Copyright

© The Author(s) 2024.
